# Salivary microbiome and metabolome analysis of severe early childhood caries

**DOI:** 10.1186/s12903-023-02722-8

**Published:** 2023-01-19

**Authors:** Kai Li, Jinmei Wang, Ning Du, Yanjie Sun, Qi Sun, Weiwei Yin, Huiying Li, Lingqiang Meng, Xuecong Liu

**Affiliations:** 1 Department of Stomatology, Children’s Hospital of Hebei Province, Hebei Medical University, Shijiazhuang, China; 2 Department of Prosthodontics, Hospital of Stomatology Hebei Medical University, Hebei Medical University, Shijiazhuang, China

**Keywords:** SECC, Saliva, Microbiome, Metabolome, Biomarkers

## Abstract

**Background:**

Severe early childhood caries (SECC) is an inflammatory disease with complex pathology. Although changes in the oral microbiota and metabolic profile of patients with SECC have been identified, the salivary metabolites and the relationship between oral bacteria and biochemical metabolism remains unclear. We aimed to analyse alterations in the salivary microbiome and metabolome of children with SECC as well as their correlations. Accordingly, we aimed to explore potential salivary biomarkers in order to gain further insight into the pathophysiology of dental caries.

**Methods:**

We collected 120 saliva samples from 30 children with SECC and 30 children without caries. The microbial community was identified through 16S ribosomal RNA (rRNA) gene high-throughput sequencing. Additionally, we conducted non-targeted metabolomic analysis through ultra-high-performance liquid chromatography combined with quadrupole time-of-flight mass spectrometry to determine the relative metabolite levels and their correlation with the clinical caries status.

**Results:**

There was a significant between-group difference in 8 phyla and 32 genera in the microbiome. Further, metabolomic and enrichment analyses revealed significantly altered 32 salivary metabolites in children with dental caries, which involved pathways such as amino acid metabolism, pyrimidine metabolism, purine metabolism, ATP-binding cassette transporters, and cyclic adenosine monophosphate signalling pathway. Moreover, four in vivo differential metabolites (2-benzylmalate, epinephrine, 2-formaminobenzoylacetate, and 3-Indoleacrylic acid) might be jointly applied as biomarkers (area under the curve = 0.734). Furthermore, the caries status was correlated with microorganisms and metabolites. Additionally, Spearman's correlation analysis of differential microorganisms and metabolites revealed that *Veillonella*, *Staphylococcus*, *Neisseria*, and *Porphyromonas* were closely associated with differential metabolites.

**Conclusion:**

This study identified different microbial communities and metabolic profiles in saliva, which may be closely related to caries status. Our findings could inform future strategies for personalized caries prevention, detection, and treatment.

**Supplementary Information:**

The online version contains supplementary material available at 10.1186/s12903-023-02722-8.

## Background

Dental caries is among the most common chronic childhood diseases, affecting > 560 million children worldwide [1]. Early childhood caries (ECC) is defined as the presence of caries in children aged < 6 years of age. Further, severe ECC (SECC) is defined as any sign of smooth-surface caries in children < 3 years of age, and from ages 3 through 5, one or more cavitated, missing (due to caries), or filled.

smooth surfaces in primary maxillary anterior teeth or a decayed, missing, or filled score of ≥ 4 (age 3), ≥ 5 (age 4), or ≥ 6 (age 5) surfaces constitute S-ECC [2]. The prevalence of ECC in developed and developing countries is 1–12% and up to 70%, respectively [3]. Additionally, ECC is more prevalent in lower social income groups [4, 5]. A Chinese oral epidemiological survey conducted in 2018 found that the prevalence of dental caries in the milk teeth of 5-year-old children was 71.9%, which indicated a ≈ 6% increase compared with that reported 10 years ago; further, the untreated rate was as high as 95.9% [6].

The severe effects of SECC on masticatory function may cause height and weight deficits in children [7], which results in various adverse physical and psychological effects. Moreover, SECC reduces the overall quality of life and imposes a huge economic burden on families and society [8, 9]. Therefore, there is a need to elucidate the underlying mechanism and develop relevant biomarkers for early personalized prevention, diagnosis, and treatment of caries [10].

The oral microbiota is among the most complex microbiotas in the human body, with > 700 bacterial species present [11]. Due to the limitations of microbiological research, *Streptococcus mutans* and *Lactobacillus* have been long considered the specific pathogens for ECC. However, from an ecological perspective, ECC is now considered to arise when environmental disturbances alter the oral microbiota balance. Eventually, caries-causing bacteria predominate, resulting in demineralization and decomposition of dental tissue [12–14].

Variations in oral microbiota among different ecological niches as well as interactions within and outside the host during ECC development remain unclear. Saliva is considered an important medium for reflecting individual oral microbial characteristics and various disease states [15]. There are significant differences in the salivary microbial community between caries hosts and caries-free hosts [16, 17], with several studies exploring possible biomarkers [18–20]. The application of metabolomics techniques has facilitated the identification of small molecule metabolites that partly reflect the metabolic profile of the flora and are used to identify disease-related biomarkers. Metabolomics techniques have recently become increasingly sophisticated and have been used in studies on dental caries [21], periodontitis [22], and oral cancer [23]. However, only a few studies have investigated childhood caries, mainly involving plaque [24] and saliva [25]. A study on the salivary nuclear magnetic resonance (NMR) metabolome of children under different conditions suggested that non-stimulated salivary metabolomics may present the metabolite profile of caries [26]. However, most studies have conducted independent microbiome analyses. Additionally, although several studies have demonstrated differences in flora according to the disease states, the microbial interactions remain unclear.

To our knowledge, no studies have applied a multi-omics approach to explore salivary microbial interactions in the caries state. We aimed to identify microbial communities and metabolic profiles in children with and without SECC by combining high-throughput sequencing of 16S ribosomal RNA (rRNA) genes and untargeted metabolomics through ultra-high performance liquid chromatography combined with quadrupole time-of-flight mass spectrometry (UHPLC-Q/TOF–MS). Additionally, we aimed to explore salivary biomarkers for caries status and the possible mechanisms of microbial interactions in order to inform future strategies for the prevention and diagnosis of caries in children.

## Materials and methods

### Study population and clinical examination

This study was approved by the Ethics Committee of Hebei Children's Hospital (No. 207). All legal guardians of participating children were provided written informed consent following the Declaration of Helsinki. In June 2020, the Department of Stomatology of Hebei Children's Hospital enrolled 60 children in a kindergarten under the jurisdiction of Shijiazhuang, including 30 children with SECC (SECC group) and 30 children without caries (Group CF). The inclusion criteria were as follows: local kindergarten students in Shijiazhuang, no history of long-term (> 3 months) relocation; no use of antibiotics, antibacterial mouthwash, or toothpaste use within 1 month; no orthodontic devices; no systemic diseases; no oromandibular system abnormalities and salivary gland diseases; and no irritable or restless behaviour during examination or sample collection. A single physician clinically examined the caries status of the children under natural light based on the World Health Organization guidelines and records regarding the child's sex, age, ethnicity, caries status, etc.

#### Sample collection

The participants and their guardians were instructed not to perform oral care (brushing and flossing) in the morning on the day of sample collection and not to eat or drink for 2 h before sample collection. Sample collection was performed in the morning (9:30 a.m. to 10:00 a.m.) by four paediatric dentists and six kindergarten teachers (for emotional reassurance of young children). Specifically, after mouth rinsing with distilled water, approximately 3 mL of non-irritating saliva was collected in a quiet state using a sterile 50 ml centrifuge tube. Subsequently, samples were stored in two separate 1.5 mL Eppendorf tubes using sterile pipettes, immediately placed in an insulated box filled with dry ice, and transported to the laboratory for storage at -80 °C before further processing. One sample was used for 16SrRNA sequencing and the other for metabolic assessment. The successful sample collection rate was 100%.

### Sample preparation and 16S rRNA gene sequencing

#### Genomic DNA extraction and PCR amplification

The genomic DNA of the samples was first extracted using the CTAB/SDS method, the DNA purity and concentration were checked using agarose gel electrophoresis. DNA was diluted as per the concentration l μg/μL using sterile water.

Using the diluted genomic DNA as template, the 16S V3-V4 sequencing region was selected and PCR was performed using specific primers (341F CCTAYGGGRBGCASCAG and 806R GGACTACNNGGGTATCTAAT). All PCR reactions were carried out with 15µL of Phusion® High-Fidelity PCR Master Mix (New England Biolabs). The PCR reaction procedure was as follows: 98 °C pre-denaturation for 1 min; 30 cycles including (98 °C, 10 s; 50 °C, 30 s; 72 °C, 30 s); 72 °C, 5 min.

#### Mixing and purification of PCR products

The PCR products were detected by electrophoresis using 2% concentration of agarose gel; the resulting products were purified by magnetic beads, quantified by enzyme labelling, and then mixed in equal amounts according to the concentration of PCR products. Then, they were mixed thoroughly and detected by electrophoresis using a 2% agarose gel. The products were recovered using Qiagen gel recovery kit (Qiagen, Germany) for the target bands.

#### Library construction and sequencing

Libraries were constructed using TruSeq® DNA PCR-Free Sample Preparation Kit (Illumina, USA) library construction kit, and the constructed libraries were quantified by Qubit® 2.0 Fluorometer (Thermo Fisher, USA) and Q-PCR; subsequently, the libraries were sequenced using NovaSeq6000 (Illumina, USA) and 250 bp paired-end reads were generated. Sequence are processed using the Tags quality control process from QIIME (Version 1.9.1, http://qiime.org/scripts/split_libraries_fastq.html). The UPARSE algorithm was applied to analyze the sequences (UPARSE v7.0.1001, http://www.drive5.com/uparse/). Sequences with ≥ 97% similarity were assigned to the same operational taxonomic units (OTUs). The abundance information of the OTUs was normalized using a standard of sequence number corresponding to the sample with the least number of sequences. The subsequent computation of alpha and beta diversities was performed using QIIME (Version 1.9.1) (Additional file 1).

### Metabolome sample preparation and testing conditions

#### Sample preparation

The samples were sent to the laboratory for centrifugation at 13,500 r/min at 4 °C for 10 min. The supernatant was removed, dispensed and stored at -80 °C. The samples were removed from the -80 °C refrigerator at the beginning of the experiment and thawed; 100 μL of acetonitrile was added to 50 μL of the saliva sample, vortexed for 30 s at 15,000 r/min, and centrifuged at 4 °C for 10 min; this process was performed again. The supernatant was used for analysis. Three different types of samples were used during the sample analysis, including the blank solution, quality control sample, and real sample. The injection order of the samples can have a significant impact on the experimental results. Therefore, the injection sequence in each model was varied. The blank solution and QC sample were sequentially injected five times and six times, respectively. After that, the random sampling method was performed in the real sample analysis process and one blank solution and one QC sample were inserted into every eight real samples. The blank solution was a 95% acetonitrile solution to balance the system. The QC was used to evaluate the precision of the instrument before the analysis process and to evaluate whether the experimental condition was stable from the first real sample to the last one in each analysis model. It was separately prepared, pooled, and separately mixed with the same volume of six randomly selected processed real samples.

#### Testing conditions

An AB SCIEX Q-TOF 5600 + triple quadrupole-time of flight mass spectrometer with Shimadzu LC-30A ultra performance liquid chromatograph (Kyoto, Japan) and Triple-TOFTM5600 + mass spectrometer (AB SCIEX, USA) was used. Since saliva contains approximately 99% water, many endogenous metabolites were expected to be highly polar. HILIC columns have excellent separation capabilities for the analysis of strongly polar endogenous substances, while HSS T3 columns target substances with low to medium polarity. The liquid phase part was separated using a hydrophilic (HILIC) column and a reversed-phase (HSS T3) column, and the mass spectrometry part was acquired in full scan mode using an ESI source in the positive ion mode. The experiments were divided into two modes HILIC ( +) mode and HSS T3 ( +) mode. The hydrophilic column was ACQUITY UPLC® BEH HILIC (2.1 × 100 mm, 1.7 μm), and the mobile phases included 10 mM aqueous ammonium acetate (A) and acetonitrile (B) with gradient elution. The elution procedure was as follows: 0–2 min, 95–95% B; 2–8 min, 95–75% B; 8–9.5 min, 75–55% B; 9.5–10 min, 55–95% B; 10–15 min, 95–95% B; flow rate: 0.3 mL/min; column temperature: 35.00 °C; injection volume 5 μL; sample chamber temperature: 4 °C. The reversed-phase column was an ACQUITY UPLC® HSS T3 (2.1 × 100 mm, 1.8 μm) with the mobile phases of 1‰ formic acid-5 mM aqueous ammonium acetate (A) and acetonitrile (B), and the gradient elution program was as follows: 0–2 min, 10–50%; 2–9.5 min, 50–95% B; 9.5–10 min, 95–10% B 10–15 min, 10–10% B; flow rate: 0.3 mL/min; column temperature: 35.00 °C; injection volume 5μL; sample chamber temperature: 4 °C.

Mass spectrometry conditions in positive ion mode: ion source, ESI source; full scan mode acquisition. MS1 conditions: acquisition range, 100–1000 Da; nebulizing gas (Gas 1), 55 psi; heating gas (Gas 2), 55 psi; curtain gas (CUR), 35 psi; temperature (TEM), 550 °C; source injection voltage (IVF), 5500 V; declustering Information-dependent acquisition (IDA): MS2 acquisition of the eight most responsive peaks above 50 cps, with dynamic background subtraction (DBS) turned on. MS2 conditions: acquisition range, 50–1000 Da; DP, 50 V; CE, 30 eV; collision energy expansion (CES), 15 eV. The experimental procedure was performed using automatic calibration (CDS).

### Statistical analysis

The 16S rRNA sequence data were analysed using the QIIME software package (Version 1.9.1) to calculate the Ace, chao1 and Shannon indices for assessing alpha diversity. Analysis of variance was performed using Student’s t-test. Additionally, cumulative box plots of species were plotted using R software (Version 2.15.3). Additionally, beta-diversity analysis was performed using R software to plot weighted/unweighted UniFrac distance metrics, principal coordinate analysis (PCoA) plots, and nonmetric multidimensional scaling (NMDS) plots based on operational taxonomic unit (OTU) levels. PCoA was performed using the WGCNA, stats, and ggplot2 packages of R software. Further, NMDS analysis was performed using the vegan package of R software. Metastats analysis was conducted using R software at each classification level (Phylum, Class, Order, Family, Genus, Species) through between-group permutation tests to obtain *p* values, which were visualized as violin plots. We performed analysis of similarity (ANOSIM), multi-response permutation procedure (MRPP), and ADONIS (permutational multivariate analysis of variance) analysis using the R vegan package's anosim function, mrpp function, and adonis function. R software based on the analysis of species abundance can be used to perform a random forest model, Significant species were screened by MeanDecreaseAccuracy and MeanDecreaseGin, after which cross-validation (default tenfold) was done for each model and ROC curves were plotted.

The raw data for the MS downcomers were acquired using Analyst TF 1.6 software (AB SCEIX, USA) and converted to mzML format through ProteoWizard using the XCMS program (http://www.bioconductor.org/packages/release/bioc/html/ xcms.html). We performed peak extraction, alignment, and retention time correction. Peak areas were corrected using the "SVR" method; moreover, peaks with > 50% deletion rate in each sample group were filtered. After calibration and filtering, peaks were identified by querying the laboratory's database, integrating public libraries, and mtDNA method. All statistical analyses, which included univariate and multivariate statistical analyses, were conducted using R software (Version 2.15.3). Univariate statistical analysis included multiplicative analysis of variance while multivariate statistical analysis included principal component analysis (PCA) and orthogonal partial least squares discrimination analysis (OPLS-DA). The quality of the OPLS-DA was evaluated by the values of R^2^X or R^2^Y and Q^2^.We performed differential metabolite enrichment analyses using the KEGG database (KEGG, http://www.genome.jp/kegg) and MetaboAnalyst 3.0 (Montreal, QC, Canada). Values for the area under the curve (AUC) of the ROC were used to assess the diagnostic utility of candidate metabolites for SECC.

We analysed the relationships among microbial communities, metabolites, and clinical indicators through Spearman’s correlation analysis and drew the heat maps. Spearman was performed with R software (R package = psych).

## Results

### Microbial profiles of saliva samples

The physiological indicators including age and sex of the participants are known to affect experimental results. The Wilcoxon Rank Sum test and Chi-squared test were used to access whether age and sex affected the experimental grouping result. There were no significant between-group differences in age or sex (Table 1).

**Table 1:** Demographic and clinical characteristics of the subjects

Clinical parameters	SECC	Caries-free
Age (month)^a^	58.57 ± 9.58	51.63 ± 9.95
Sex^b^		
Male	16	17
Female	14	13
Caries status		
dmft	8.83 ± 2.63	0
dmfs	14.83 ± 4.44	0

^a^Represented as mean ± standard deviation, No significance between SECC

group and caries-free group (*p* > 0.05), by Wilcoxon Rank Sum test

^b^No significance between SECC group and caries-free group (*p* > 0.05), by

Chi-squared test

A total of 120 saliva samples were collected, with 60 being for 16S rRNA gene sequencing. A total of 3,871,616 high-quality sequences were obtained, and the average number for each sample was 62,527. Sequence clustering yielded 2877 OTUs, which involved 42 phyla, 90 classes, 190 orders, 300 families, and 513 genera.

**Fig. 1:**
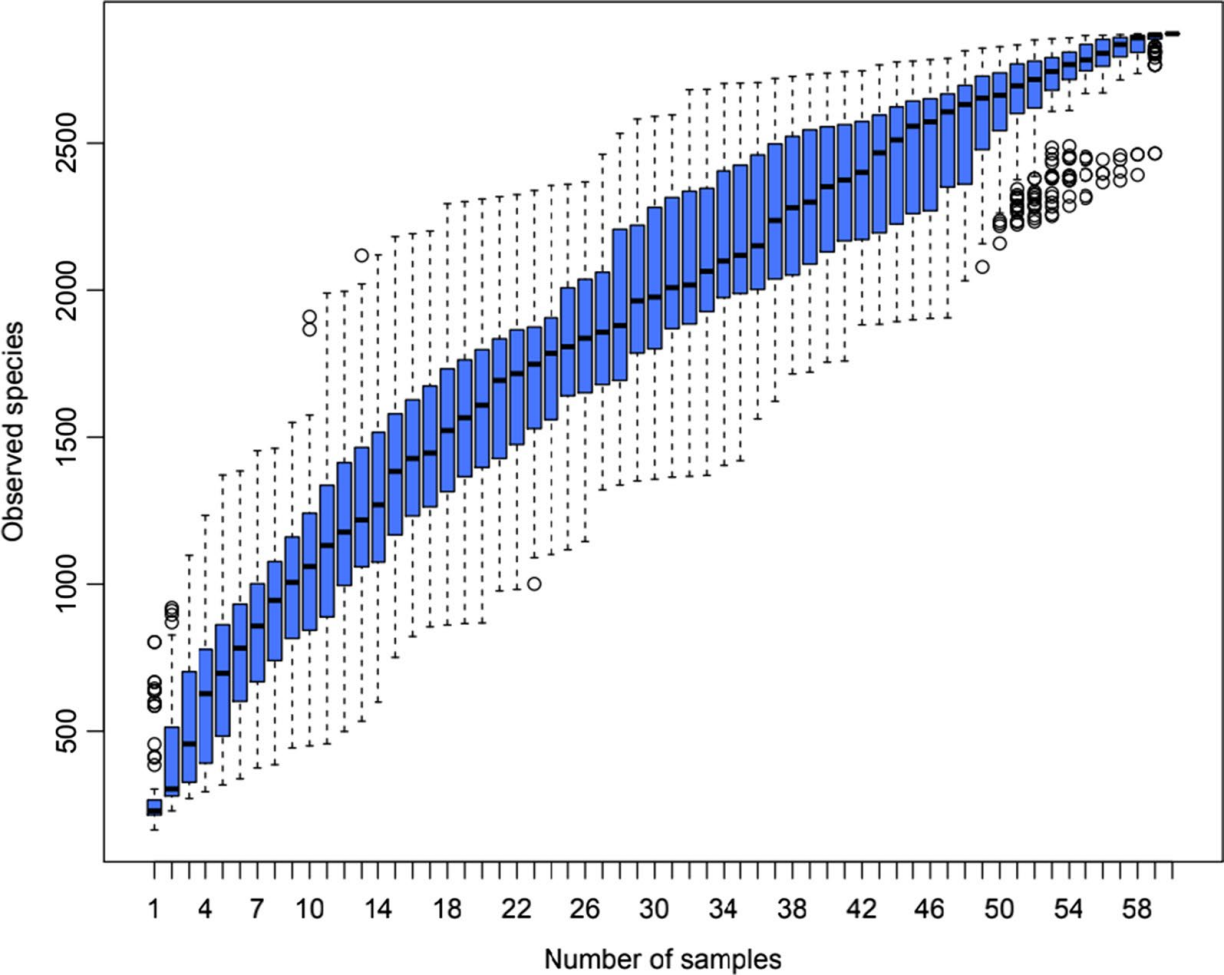
Species Accumulation Box Plot for Children with and without SECC

The species accumulation box plot reflects the rate of emergence of new OTUs (new species) with continuous sampling. The box plot position levelled off with increasing sample size, which indicated that the sampling depth could reflect the flora of salivary microorganisms (Fig. 1).

**Fig. 2:**
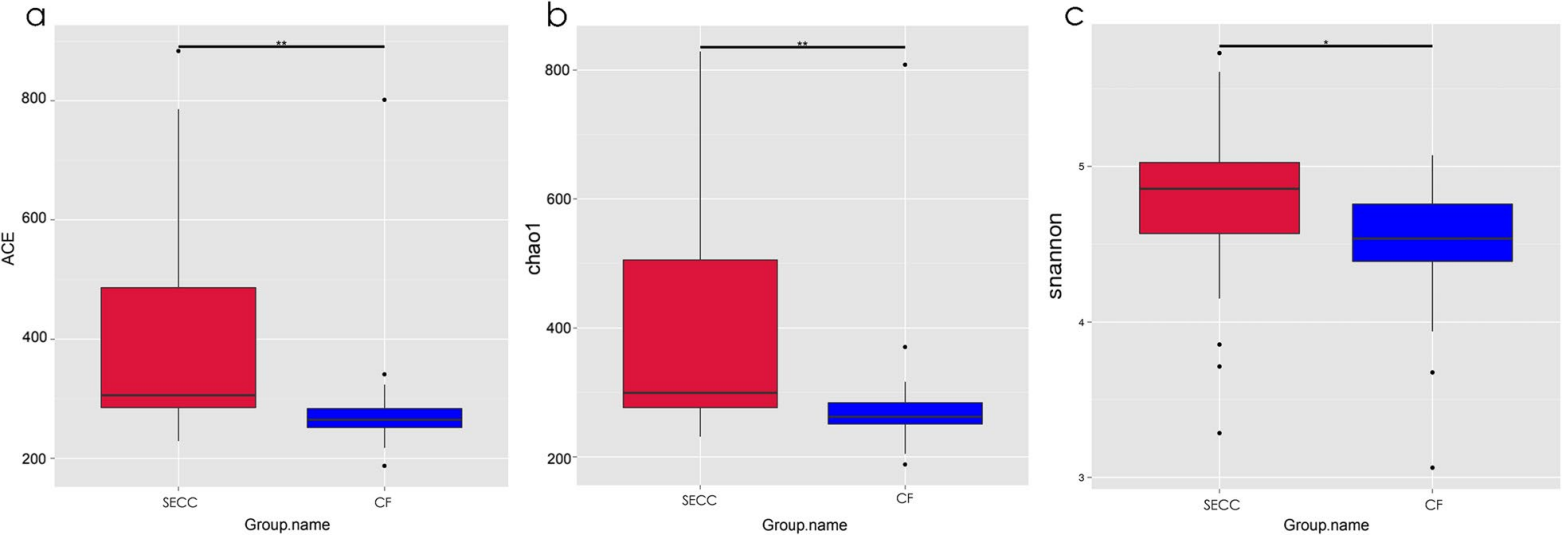
Alpha-diversity of bacterial communities of severe early childhood caries (SECC) and caries-free subjects (CF). Microbiota alpha-diversity as calculated by abundance-based coverage estimator (ACE) index (**a**), chao1 index (**b**) and shannon index (**c**) of saliva samples in both groups. *p < 0.05, **p < 0.01

**Fig. 3:**
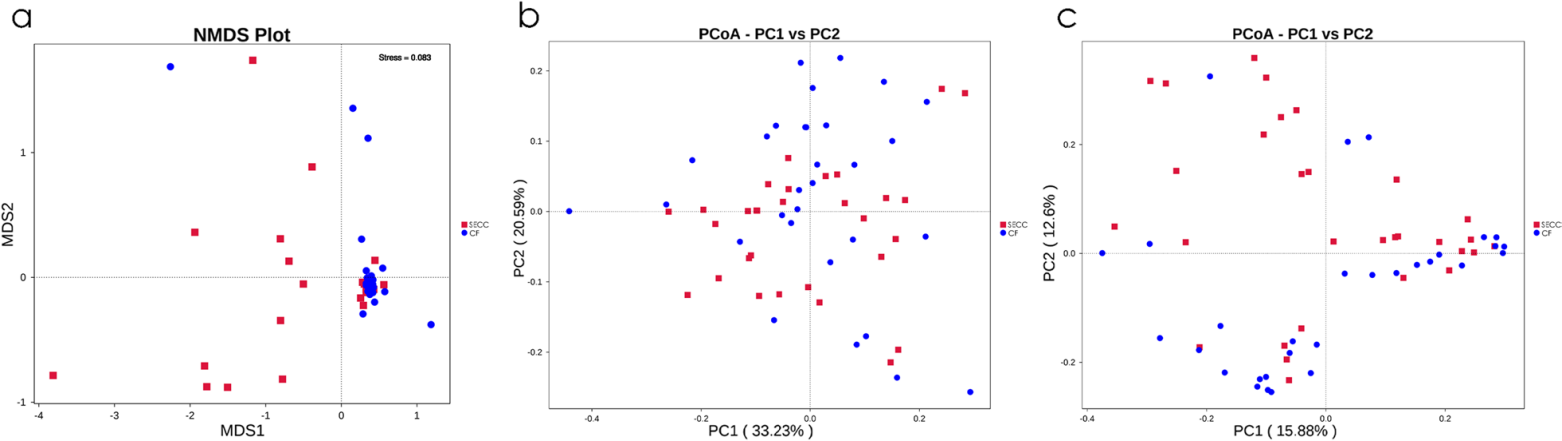
Beta-diversity of saliva samples in two groups. Nonmetric multidimensional scaling (NMDS) (**a**), as well as principal coordinates analysis (PCoA) of the unweighted (**b**) and weighted UniFrac distance (**c**), were performed based on the operational taxonomic unit (©) abundances

We used the alpha and beta diversity of the microbial community to further analyse its overall compositional richness and structural characteristics. Compared with the CF group, the SECC group showed significantly larger Alpha-diversity indices, including the abundance-based coverage estimator (ACE, *p* < 0.01), chao1 index (*p* < 0.01) and Shannon index (*p* < 0.05), which indicated a higher richness and diversity of salivary microbial communities (Fig. 2a–c). Beta-diversity analysis using OTUs in NMDS analysis, as well as PCoA analysis based on the weighted and unweighted Unifrac distance, revealed between-group differences in microorganisms (Fig. 3a–c). Moreover, the non-parametric statistical methods, including ANOSIM, ADONIS, and MRPP, revealed significant between-group differences in the overall biotope structures (all *p* < 0.05).

**Fig. 4:**
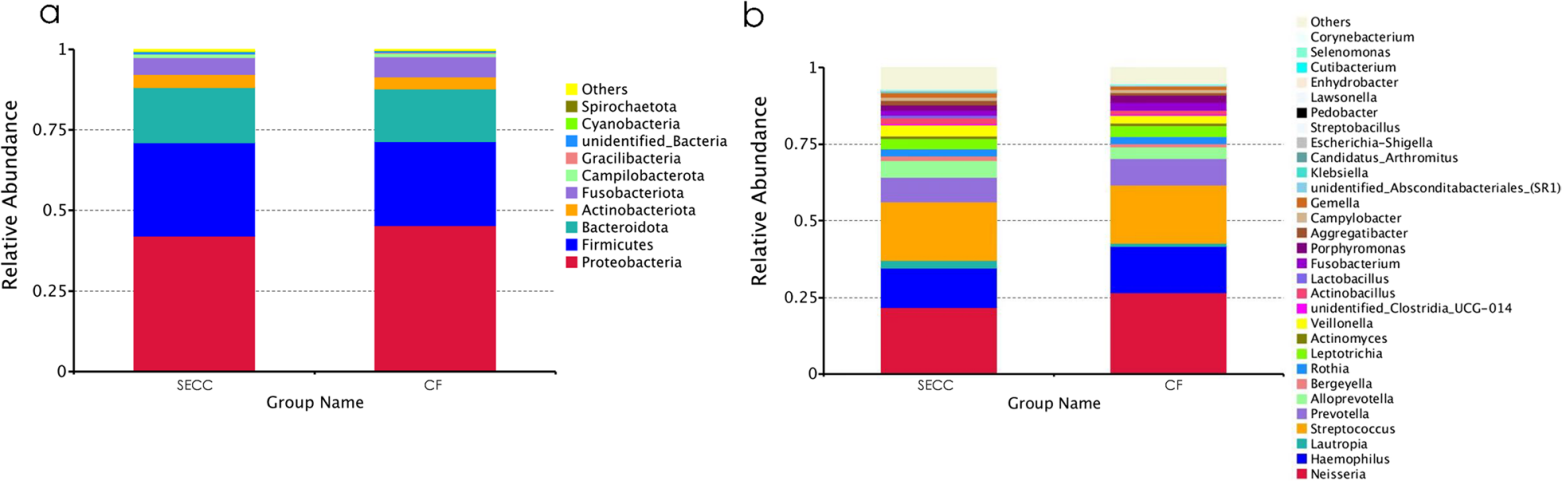
Bacterial compositions in the SECC and CF groups. Relative abundance of bacterial composition at the phylum level (**a**) and genus level (**b**) of saliva samples in the SECC and CF groups

The five most abundant species under both groups at the phylum level were *Proteobacteria*, *Firmicutes*, *Bacteroidota*, *Actinobacteriota*, and *Fusobacteriota*, which accounted for > 97% of the total sequences (Fig. 4a).

The top five most abundant species in both groups at the genus level were *Neisseria*, *Streptococcus*, *Haemophilus*, *Prevotella*, and *Alloprevotella* which accounted for > 64% of the total sequences (Fig. 4b).

Regarding Metastats analysis at the phylum and genus levels, there were between-group differences in the abundance of 8 phyla and 32 genera (relative abundance > 0.01%).

At the phylum level, the relative abundance of *Firmicutes*, *Cyanobacteria*, *Acidobacteriota*, *Methylomirabilota*, *Chloroflexi*, *Gemmatimonadetes*, and *Myxococcota* was significantly higher in the SECC group than in the CF group. Moreover, the relative abundance of *Gracilibacteri* was significantly higher in the CF group than in the SECC group (Additional file 2: Fig. S1).

**Fig. 5:**
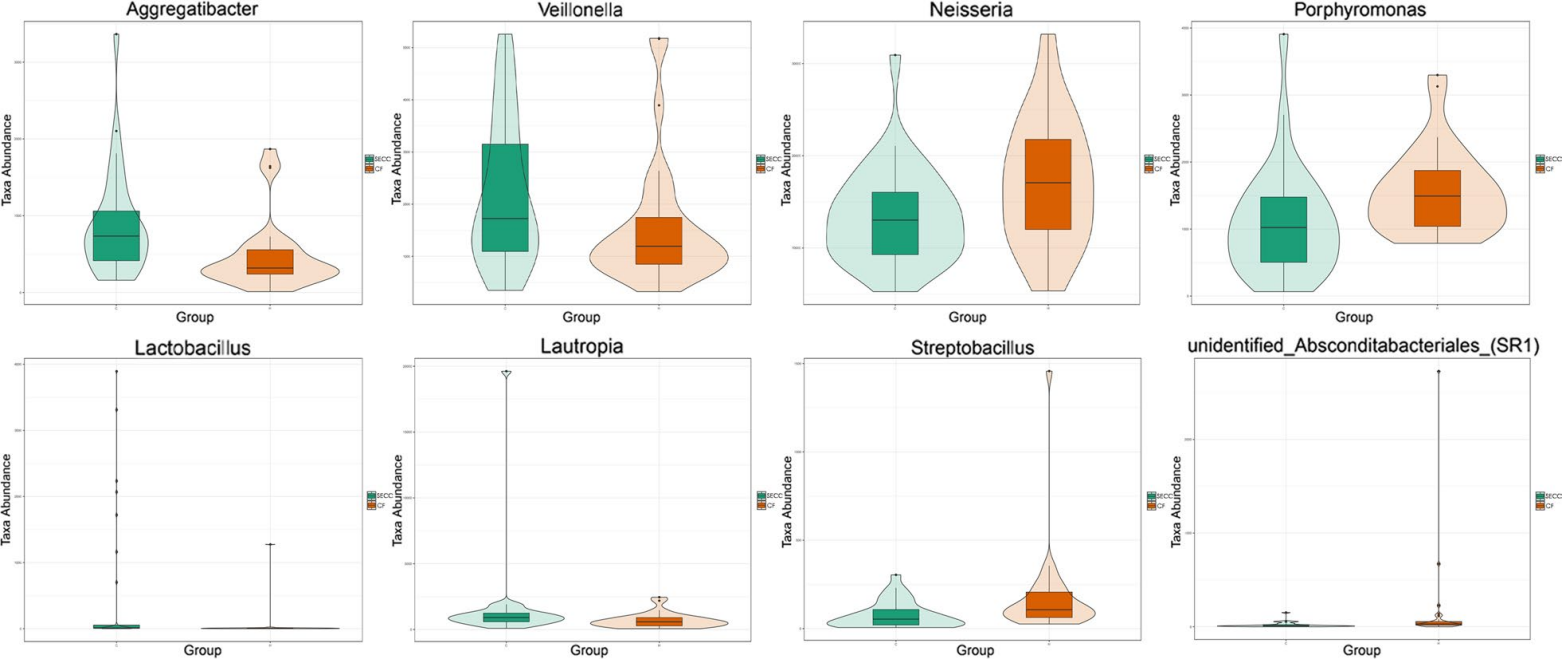
Taxon abundances at the genus levels were compared between the SECC and CF groups using Metastats. The violin plots show the top eight genera with significant between-group differences

The top eight taxa with the highest between-group differences in abundance at the genus level are presented as violin plots to visualize the distribution characteristics of the data. *Lautropia*, *Veillonella*, *Lactobacillus*, and *Aggregatibacter* were significantly enriched in the SECC group than in the CF group. Contrastingly, *Neisseria*, *Porphyromonas*,*unidentified_Absconditabacteriales_(SR1)*, and *Streptobacillus* were lower in the SECC group than in the CF group (Fig. 5).

**Fig. 6:**
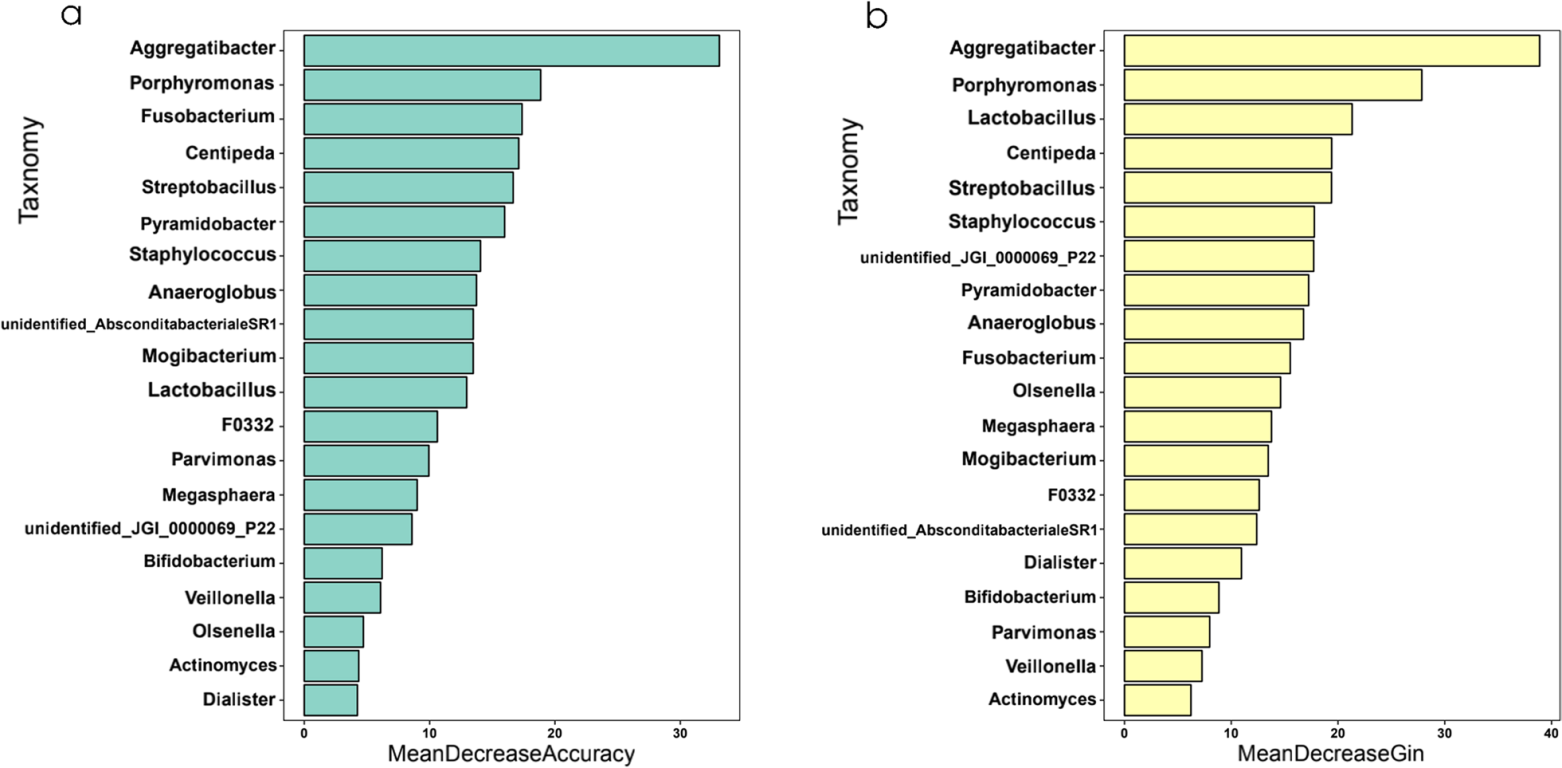
The random forest model was constructed for the genus taxonomic level. The important 20 species were screened by MeanDecreaseAccuracy (**a**) and MeanDecreaseGin (**b**)

For further sample analysis using a random forest machine learning approach, we constructed a prediction model based on two parameters (MeanDecreaseAccuracy and MeanDecreaseGin20; Fig. 6a, b). The experimental analysis was performed at genus level including 513 genera. MeanDecreaseAccuracy is the degree of reduction in the predictive accuracy of a random forest by taking the value of a variable and turning it into a random number ( larger values indicate a greater importance of the variable.) MeanDecreaseGin calculates the effect of each variable on the heterogeneity of observations at each node of the classification tree, and thus compares the importance of the variables (larger values indicate a greater importance of the variable) to filter out important species. Additionally, we verified that the maximum AUC was 85.71% when 20 microorganisms were selected, which allowed satisfactory between-group distinction (Additional file 3: Fig. S2).

### Shifts in the metabolomic profiles of saliva samples

**Fig. 7:**
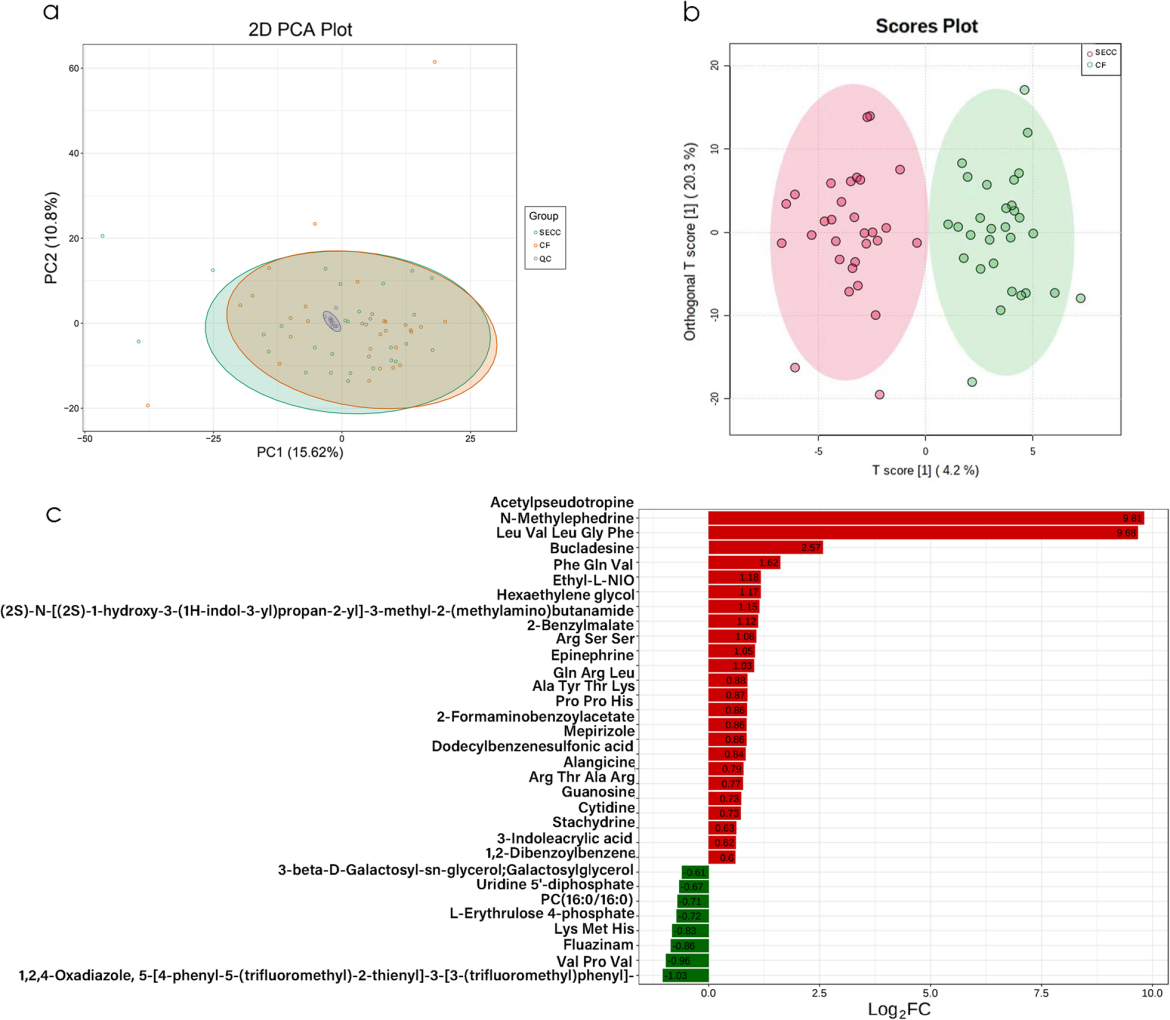
Between-group differences in the metabolic profiles. Principal component analysis (PCA) (**a**) and OPLS-DA analysis (**b**) of the salivary metabolic profiles. Values of log2 (FC) after between-group comparison (**c**). Positive and negative Log2 (FC) values indicate a relatively higher concentration in the SECC and CF groups, respectively

**Fig. 8:**
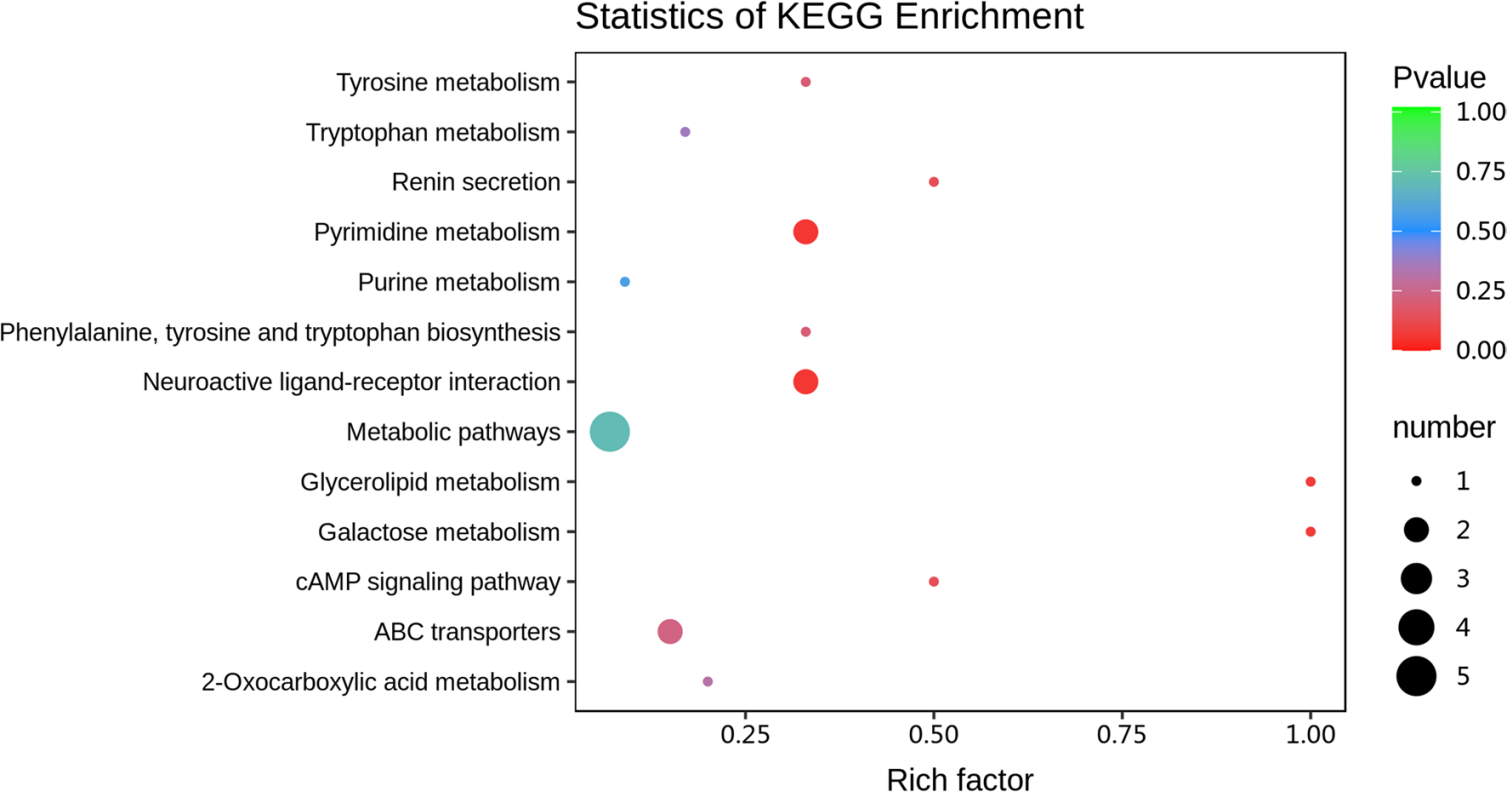
KEGG enrichment plots of the differential metabolites. The rich factor represents the ratio of the number of differential metabolites in the corresponding pathway to the total number of metabolites annotated in the pathway, with larger values indicating greater enrichment. A smaller *p* value indicates a more significant enrichment

To investigate changes in salivary metabolomics under SECC and their relationship with microbial changes, we performed untargeted metabolomics using 60 saliva samples. A total of 356 qualifiable metabolites were used in the subsequent analysis after removing internal standards and false positive peaks as well as combining peaks of the same metabolites. The experimental, control, and quality control samples in the PCA showed good aggregation. Moreover, the pre-treatment and experimental conditions for each shot were stable, which indicated that the sample data was reliable in this analytical mode (Fig. 7a). There was a clear between-group distinction in the model established using OPLS-DA; moreover, evaluation of the model quality using the 200 permutation test revealed reliable prediction and modelling ability (Additional file 4: Fig. S3), with significant alterations of the metabolic substances in the SECC group (Fig. 7b). A total of 32 differential metabolites were yielded in the HILIC (+) and HSS T3 (+) analysis models based on the criteria of a fold change ≥ 1.5 or ≤ 0.67 and VIP > 1. Among the differential metabolites, 24 and 8 were significantly upregulated and downregulated, respectively (Fig. 7c). Cytidine, 3-indoleacrylic acid, 2-formaminobenzoylacetate, guanosine, stachydrine epinephrine, Ala-Tyr-Thr-Lys, Arg-Ser-Ser, and Pro-Pro-His were significantly increased in the SECC group. Contrastingly, L-erythrulose 4-phosphate, galactosylglycerol, PC(16:0/16:0), Lys-Met-His, fluazinam, uridin’ 5'-diphosphate, Val-Pro-Val, and 1,2,4-oxadiazole were significantly increased in the CF group. These compounds are listed in Fig. 7c and table 2. Next, we used the KEGG database annotation to hierarchically classify the differential metabolites according to their involvement in the KEGG metabolic pathway. Subsequently, we performed an enrichment analysis of the differential metabolites in the KEGG pathways to identify the related metabolic pathways [27]. We identified several metabolic pathways of the differential salivary metabolites associated with dental caries, including tryptophan metabolism, pyrimidine metabolism, purine metabolism, ABC transporters, tyrosine metabolism, cAMP signalling pathway, renin secretion, galactose metabolism, phenylalanine, tyrosine and tryptophan biosynthesis (Fig. 8). Additionally, there was enrichment of intermediate metabolites such as epinephrine (neuroactive ligand-receptor interactions, renin secretion), guanosine (ABC transporters, purine metabolism), and 2-benzylmalate (phenylalanine, tyrosine and tryptophan biosynthesis, and 2-carbonylformate metabolism).

**Table 2:** Details of the differential metabolites between the SECC and CF groups

Compounds	Formula	VIP	Log2FC	Type
Acetylpseudotropine	C10H17NO2	1.35	9.81	Up
3-Indoleacrylic acid	C11H9NO2	1.98	0.62	Up
2-Formaminobenzoylacetate	C10H9NO4	2.15	0.86	Up
Cytidine	C9H13N3O5	1.66	0.73	Up
Guanosine	C10H13N5O5	2.30	0.73	Up
Stachydrine	C7H13NO2	1.07	0.63	Up
Epinephrine	C9H13NO3	1.77	1.03	Up
Ala Tyr Thr Lys	C22H35N5O7	2.12	0.87	Up
Arg Ser Ser	C12H24N6O6	2.06	1.05	Up
Pro Pro His	C16H23N5O4	2.03	0.86	Up
L-Erythrulose 4-phosphate	C4H9O7P	1.35	− 0.72	Down
Phe Gln Val	C19H28N4O5	1.87	1.18	Up
Mepirizole	C11H14N4O2	2.56	0.86	Up
3-beta-D-Galactosyl-sn-glycerol;Galactosylglycerol	C9H18O8	1.32	− 0.61	Down
N-Methylephedrine	C11H17NO	1.36	9.68	Up
Dodecylbenzenesulfonic acid	C18H30O3S	2.00	0.84	Up
Gln Arg Leu	C17H33N7O5	1.93	0.88	Up
Arg Thr Ala Arg	C19H38N10O6	2.02	0.77	Up
PC(16:0/16:0)	C40H75NO9	1.06	− 0.71	Down
Lys Met His	C17H30N6O4S1	1.51	− 0.83	Down
Leu Val Leu Gly Phe	C28H45N5O6	1.42	2.57	Up
Alangicine	C28H36N2O5	2.00	0.79	Up
2-Benzylmalate	C11H12O5	1.24	1.08	Up
Hexaethylene glycol	C12H26O7	2.16	1.15	Up
Ethyl-L-NIO	C9H19N3O2	1.90	1.17	Up
(2S)-N-[(2S)-1-hydroxy-3-(1H-indol-3-yl)propan-2-yl]-3-methyl-2-(methylamino)butanamide	C17H25N3O2	2.27	1.12	Up
Bucladesine	C18H24N5O8P	2.23	1.62	Up
Val Pro Val	C15H27N3O4	1.60	− 0.96	Down
1,2,4-Oxadiazole, 5-[4-phenyl-5-(trifluoromethyl)-2-thienyl]-3-[3-(trifluoromethyl)phenyl]-	C20H10F6N2OS	1.43	− 1.03	Down
Uridine 5'-diphosphate	C9H14N2O12P2	1.23	− 0.67	Down
1,2-Dibenzoylbenzene	C20H14O2	1.93	0.60	Up
Fluazinam	C13H4Cl2F6N4O4	1.33	− 0.86	Down

### Correlations of the microbiota and metabolites with the clinical indices for caries

**Fig. 9:**
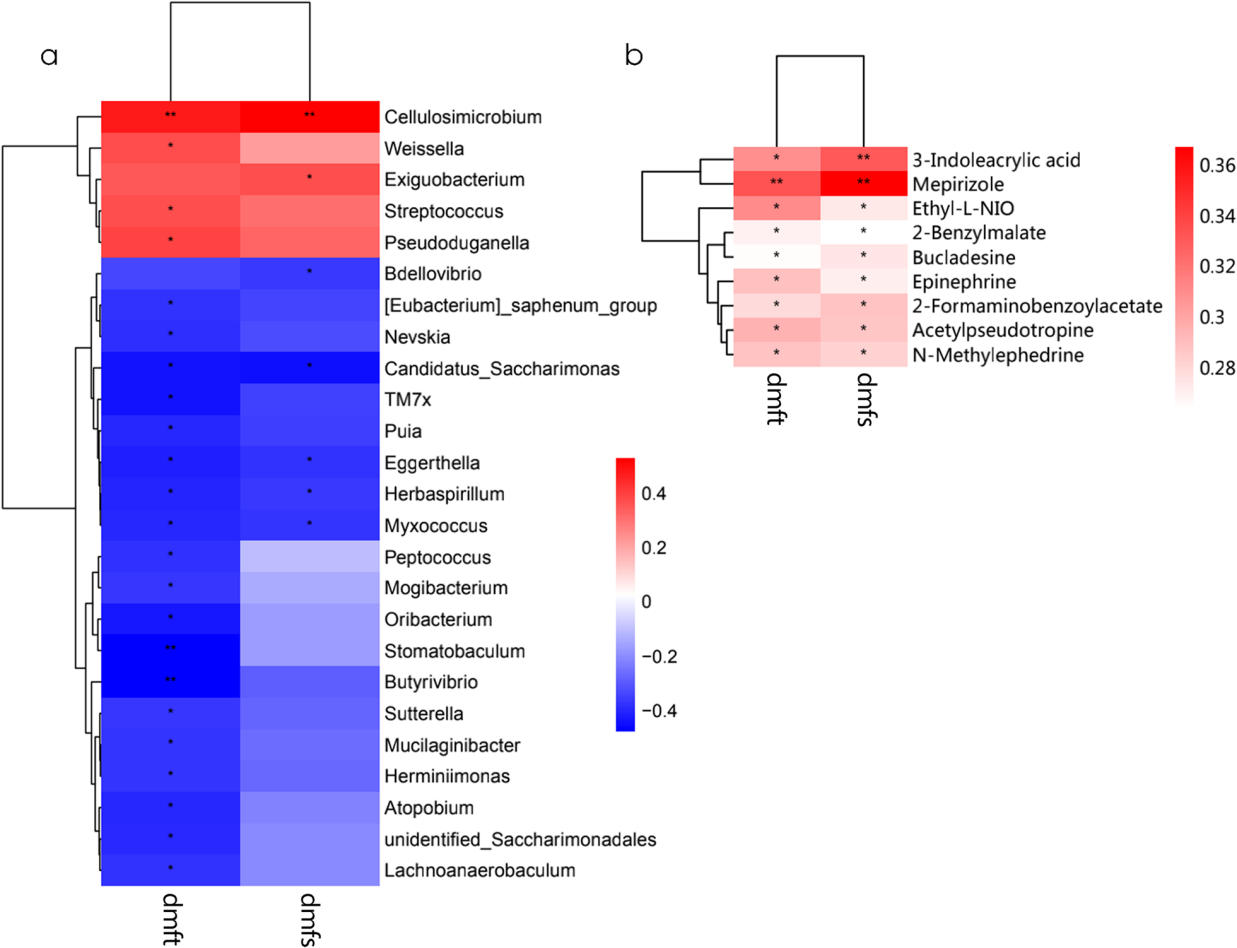
A heat map showing the correlations among the microbiota and clinical indices (only genera significantly correlated with at least one clinical index [*p* < 0.05] are shown). *Significant correlation between the genera and clinical indices (*p < 0.05, **p < 0.01) (**a**). Heat map of clinical indices correlated with differential metabolites. Spearman’s rank correlation coefficients between two clinical indices and nine differential metabolites. All nine metabolites were upregulated in the SECC group. * indicates a significant correlation between the metabolites and clinical indices (*p < 0.05, **p < 0.01) (**b**)

**Fig. 10:**
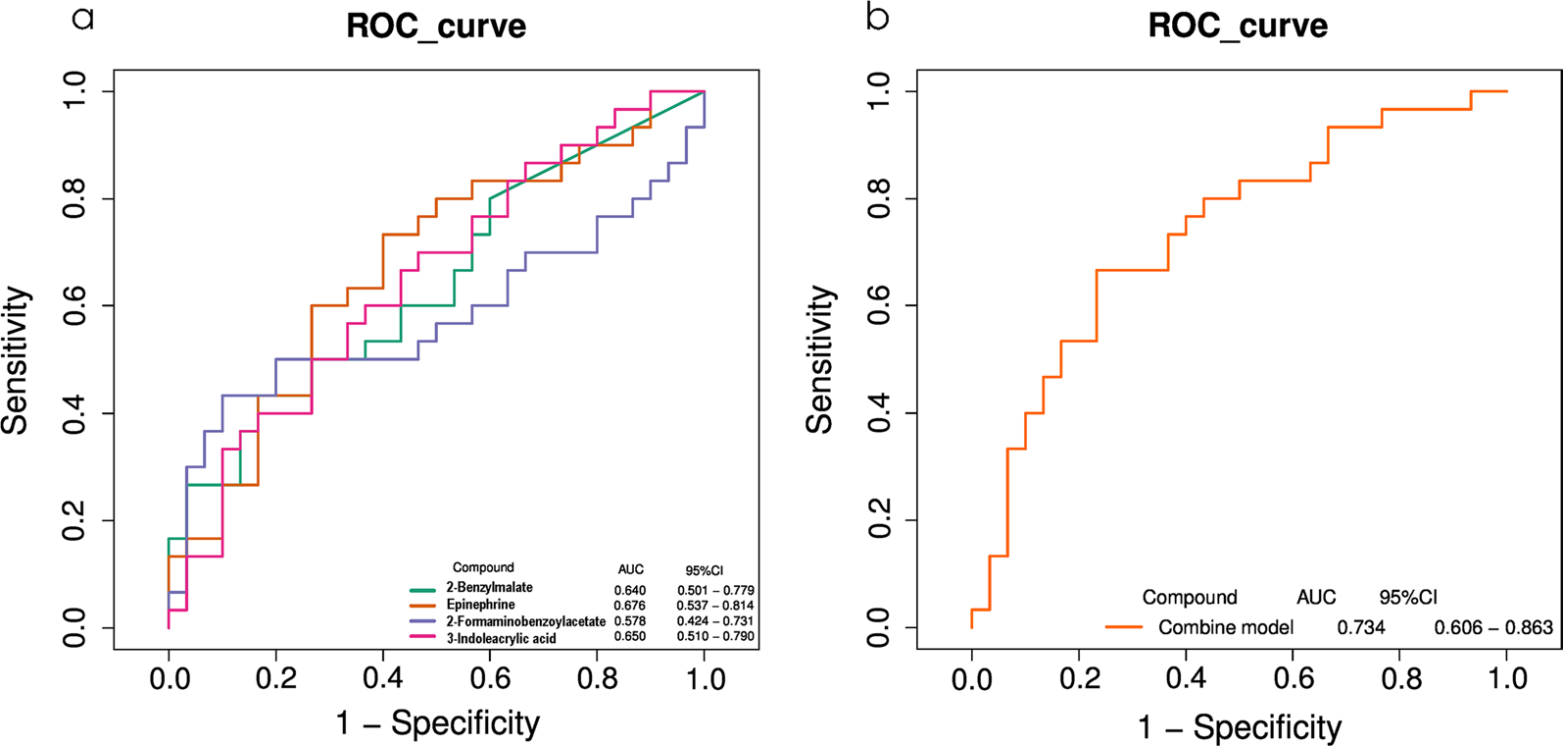
Receiver operating characteristic (ROC) curve of four differential metabolites (**a**). 2-Benzylmalate, epinephrine, 2-formaminobenzoylacetate, and 3-indoleacrylic acid were selected and validated as putative biomarkers, with an area under the curve (AUC) of 0.734 (**b**)

**Fig. 11:**
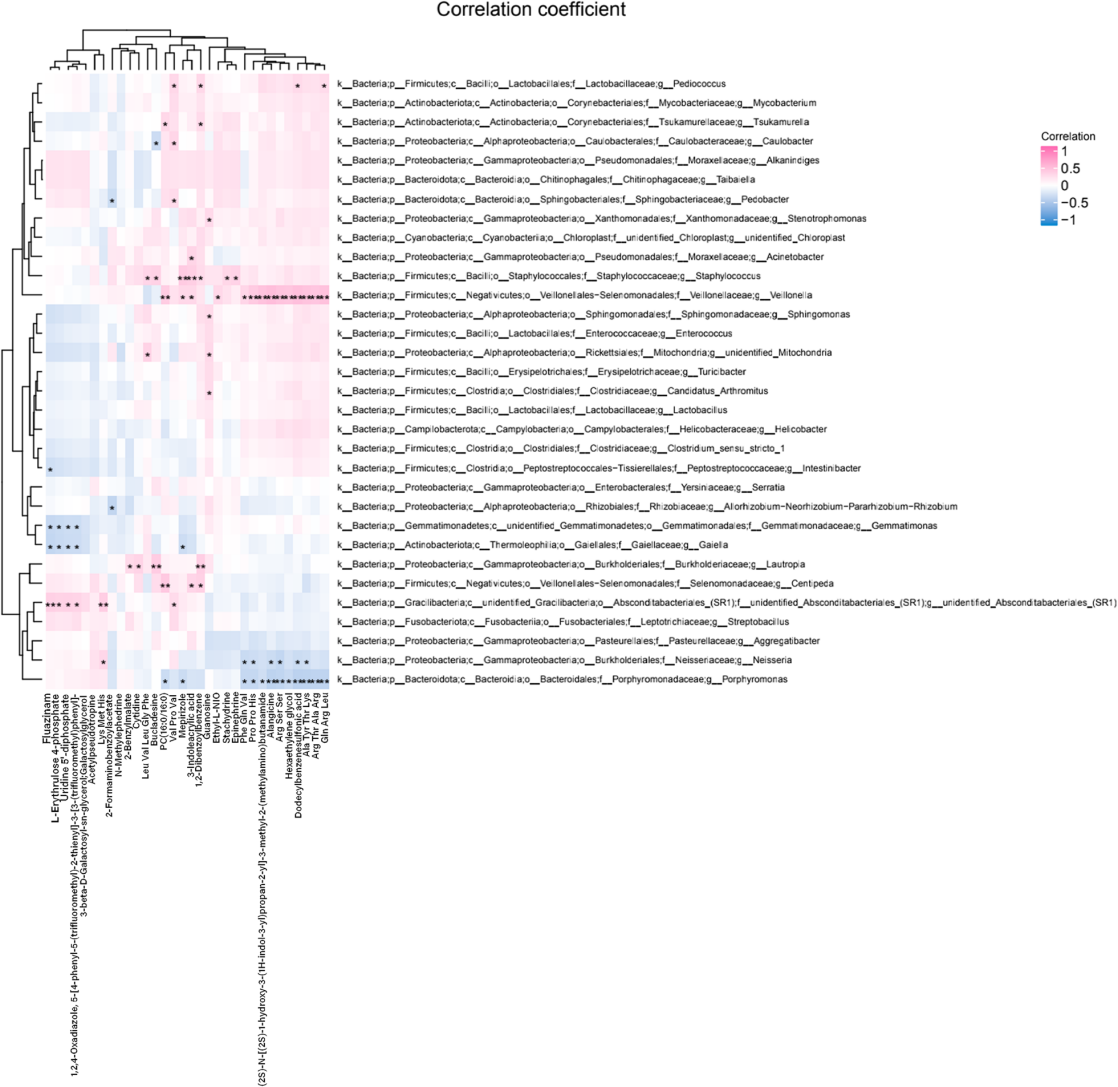
Correlations between microbiota (genus level) and metabolites in saliva. Each row and column in the graph represents a metabolite and genus, respectively, while each lattice represents a correlation coefficient between a component and a metabolite. Red and blue represent positive and negative correlations, respectively. *Indicates a significant correlation between the genera and metabolites (*p < 0.05, **p < 0.01)

We found that 25 genera were significantly correlated with at least one of the clinical datasets (Fig. 9a). Known caries-related genera, including *Streptococcus* and *Weissella*, were positively correlated with caries status, while *Eggerthella, Sutterella, Peptococcus, and Atopobium* were negatively correlated with clinical indices. These findings suggest that alterations in some salivary flora may be related to clinical indices in children. Regarding the metabolome, nine differential metabolites were positively correlated with clinical data, which suggests a close association between salivary metabolites and dental caries given the abundance and diversity of microorganisms in the SECC group (Fig. 9b). Among the aforementioned metabolites, we included four endogenous metabolites in the ROC analysis, including 2-benzylmalate, epinephrine, 2-formaminobenzoylacetate, and 3-indoleacrylic acid. They demonstrated moderate predictive power (AUC = 0.734), and thus could be potential biomarkers of the inflammatory status (Fig. 10). To further explore the correlation between salivary microbial alterations and metabolite changes, we examined the correlations between phylum and genus with between-group differences and 32 metabolites. The metabolites were correlated with five phyla, including *Gracilibacteria*, *Firmiutes*, *Acidobacteriota*, *Methylomirabilota*, and *Myxococcota* (Additional file 5: Fig. S4). Moreover, 20 genera were correlated with metabolites. Among them, *Veillonella*, *Staphylococcus*, *Neisseria*, and *Porphyromonas* showed the most extensive correlations with metabolic differentials; specifically, they were correlated with 7, 14, 7, and 13 differential metabolites, respectively. Moreover, *Veillonella* and *Staphylococcus* showed significant positive correlations with the metabolites, while *Neisseria* and *Porphyromonas* showed negative correlations with the metabolites (Fig. 11).

## Discussion

We used 16SrRNA gene sequencing and UHPLC-Q/TOF–MS untargeted metabolomics to assess and compare differential salivary microbiota and their metabolites in children with and without SECC. We screened for potential microbial and metabolite markers for caries in children and investigated the mechanisms underlying changes in the oral microbial ecosystem in SECC.

### Microbiome

Dental caries is caused by various microorganisms rather than a specific bacterium. Specifically, it is caused by a complex interaction involving at least tens of bacteria [28, 29]. Differences in the composition of oral microorganisms can distinguish the caries status and can facilitate disease diagnosis and prognosis [30–32] as well as prediction of caries occurrence [33]. However, there remains no consensus regarding cariogenic microorganisms and functional composition.

The composition of the oral microbiota changes throughout the life span from newborns to young adults with mixed and permanent dentition to the elderly [34–36]; further, it differs according to sex [37].

A study on children aged 4–6 years showed that the plaque microbiota showed increased sensitivity to the host than saliva with age progression; moreover, the oral microbiota could distinguish the different age-related changes and identify caries occurrence in these children [38]. In our study, there were no between-group differences in age and sex; moreover, all participants were from a local kindergarten and belonged to the Han ethnicity.

The oral cavity is a highly heterogeneous ecosystem with “a healthy core microbiota" in children [39]. Compared with dental plaque, saliva has more microbial functional markers since microbiota attached to teeth and soft tissue surfaces continuously flow into saliva, which makes saliva a reservoir of the entire oral microbiota [40]. The microbial and metabolic compositions and pathways differ across ecological niches. The salivary microbiota is valuable for predictive modelling and has considerable practical advantages as a sampling site, especially for children with poor compliance.

We further investigated the microbiota data through alpha and beta diversity analysis. Compared with the CF group, the SECC group showed a significantly greater alpha index (ACE, chao1, *p* < 0.01 and Shannon, *p* < 0.05) than the CF group. This is indicated that children with SECC had a higher microorganism abundance and diversity than children without SECC, which is consistent with previous reports [38, 41]. Previous studies have demonstrated that only the flora structure, but not the salivary microbial communities, differ between children with and without SECC [42–44].

These findings suggest that the appearance of dental caries may be related to oral flora disorders, which should be further investigated. The observed between-group differences in the indices of community diversity (ANOSIM, MRPP, and ADONIS; *p* < 0.05) demonstrate that the significant alterations in the structure of the salivary microbial community contributed to caries occurrence. Regarding genus classification, Metastat analysis revealed significant between-group differences in the abundance of *Neisseria*, *Lautropia*, *Lactobacillus*, *Porphyromonas*, and *Aggregatibacter* (*p* < 0.05). A previous study reported that *Neisseria* was more abundant in CF children and could be a diagnostic biomarker [45], which is consistent with our findings. Additionally, a previous study found that *Veillonella* was more abundant in childhood caries and that its co-aggregation and adhesion with *Streptococcus spp*. promotes biofilm formation and metabolic synergistic growth [46]. However, our findings regarding the differential flora are only partially consistent with previous reports [34, 47]. This could be attributed to differences in the study methodology as well as the age, ethnicity, and regions of the participants. There may be similarities in the functional performance of the combinations of different strains, which demonstrates the need for related metabolomic studies. Furthermore, most differential strains were of species with low abundance, which is consistent with previous reports [48]. This suggests the dominant flora routinely defined in the oral cavity do not comprise the microbiome biomarkers or disordered flora related to caries development.

Additional screening of the species using a random forest machine learning algorithm [49] showed that the selection of 20 bacterial species yielded the largest ROC value (85.71%). Among these species, only six had an abundance < 1%, which further demonstrates the importance of low-abundance species in saliva as markers for oral caries.

### Metabolome

To our knowledge, this is the first study to apply UHPLC-MS untargeted metabolomics to probe the salivary metabolomic profile of children with SECC and to combine this approach with microbiomics.

Metabolomic studies on caries have mainly applied NMR assays [50, 51], with only a few studies using MS [25]. NMR-based metabolomics techniques are widely used in non-targeted studies given their stability, high discrimination, and excellent reproducibility; however, NMR has an inherent disadvantage of low resolution [52].

Contrastingly, MS-based metabolomics allows highly selective and sensitive quantitative analysis, which facilitates the detection of low-molecular-weight compounds at concentrations below the range of nanogram per millilitre [53]. Additionally, since LC–MS allows optimized detection of each compound in a complex mixture, it facilitates improved separation of complex systems [54]. In our study, salivary metabolites, including amino and organic acids, were positively correlated with the bacterial load; furthermore, the oral microbiota significantly contributed to the salivary metabolome. The salivary metabolome can facilitate the diagnosis of conditions reflecting ecological dysbiosis [55]. In addition, the salivary metabolome composition is influenced by multiple physiological and environmental factors [56]. The inclusion of children as study participants allows circumvention of the effects of smoking, alcohol consumption, and complex organismal and oral environment. A previous NMR study showed that caries status, but not sex and dental stage, significantly affected the salivary metabolic profile [26]. Additionally, the salivary metabolic profile did not significantly differ between stimulated and unstimulated saliva. However, stimulation is expected to affect salivary composition since unstimulated saliva (resting state) is mainly secreted by the submandibular and sublingual glands, while stimulated saliva is mainly secreted by the parotid gland [57].

Previous metabolomic studies on bacterial plaque biofilms [24, 58] have suggested large differences in the two ectopic differential metabolites according to caries status, which is slightly inconsistent with our findings. This could be attributed to between-study differences in the ectopic flora, participants, experimental and statistical methods, saliva collection site in the oral cavity, and host circulating metabolites.

### Carbohydrate metabolism

Oral microorganisms in children with caries can metabolize intrinsic carbohydrates through various pathways. Additionally, carbohydrate metabolism is closely related to caries occurrence and development. In our study, we observed metabolite pathway analysis revealed significant enrichment of galactose metabolism. *Streptococcus mutans*, which is the main pathogen in dental caries, shows highly complex galactose utilization [59, 60]. Galactose metabolism may be used as a marker for children at a high risk of caries risk [61] and is active in the gingival crevicular fluid in patients with periodontitis [62].

During caries development, excess carbohydrate levels can alter the local microenvironment and contribute to caries induction by related bacteria such as *Streptococcus mutans* [63].

Galactose metabolism by *Streptococcus mutans* mainly occurs in the plaque. Our findings of decreased galactose metabolite levels in the SECC group are inconsistent with previous reports by NMR metabolomic studies. This could be attributed to the fact that differences in the ingested carbohydrates and/or oral habits among participants may influence the measured carbohydrate levels. Therefore, our findings regarding carbohydrate metabolism should be treated with caution.

### Organic acid metabolism

Unexpectedly, 2-benzylmalate was the only differential organic acid metabolite, which appears to be inconsistent with the acidic conditions contributing to surface demineralization of dental tissues, and thus caries production. Short-term salivary secretion may not allow sufficient accumulation of organic acids due to saliva removal as well as the saliva’s strong buffering and dilution capacity. This further demonstrates the large differences in the saliva metabolic changes within the two ecological niches. A study on the metabolic pathways involved in different oral hygiene practices suggested the involvement of 2-oxocarboxylic acid metabolism [64]. Previous studies have reported altered levels of lactate [50] and butyric acid [26], which is inconsistent with our findings. This could be attributed to differences in the experimental techniques or classes of bacteria fermentation.

### Amino acid metabolism

We did not identify any differential amino acids, which is consistent with a previous study on salivary metabolomics [65]. However, we observed enrichment of tryptophan metabolism; tyrosine metabolism; and intermediates of phenylalanine, tyrosine, and tryptophan biosynthesis processes. This could be attributed to matrix collagen degradation in the dentin during caries development [66] as well as the hydrolysis of salivary proteins/peptides by protein-hydrolysing oral bacteria [67]. In saliva, there is complex mutual facilitation between the synthesis and metabolism of tryptophan and tyrosine.

Our finding of increased metabolism of tyrosine, which is an amino acid precursor for the synthesis of catecholamines such as epinephrine, norepinephrine, and dopamine, is consistent with previous reports [26]. Moreover, disrupted tyrosine metabolism may be closely related to aggressive periodontitis [68]. Additionally, the observed increased tryptophan synthesis is consistent with previous reports [47]. Disrupted tryptophan metabolism is also related to the development of oral ulcers [69]. Contrastingly, other studies have reported decreased phenylalanine levels in children with dental caries [51]. There are significant changes in aspartic acid, ornithine, arginine, and proline metabolism related to dental caries [25]. Differences in previous reports regarding the types and pathways of amino acids in saliva involved in caries, which have also been demonstrated in studies on periodontal metabolomics [62], suggest the need to focus on changes in the amino acid metabolic pathways rather than single metabolites.

Taken together, functions related to amino acid metabolism may be crucial in the oral microecology under caries conditions, which should be further investigated.

### Other metabolic pathways and metabolites

ABC transporters mediate important substances, including carbohydrates, amino acids, proteins, lipids, and inorganic ions, crucially involved in biofilm formation [70]. The formation and maturation of plaque biofilm is a prerequisite for caries formation; accordingly, the salivary microecology undergoes changes that promote caries development.

A previous microbiomic study reported a correlation of SECC and recurrent caries with ABC transporters [71]. Accordingly, it is important to pay attention to further elucidate the role of ABC transporters in biofilm formation and function as a necessary condition for caries development.

Uridine 5'-diphosphate, cytidine, and guanosine were enriched in purine and pyrimidine metabolism, which is consistent with previous reports [26]. Moreover, a study on periodontitis reported increased hypoxanthine levels [72]. This suggests that oxidative stress and inflammation accelerate purine degradation. Pyrimidine metabolism is crucially involved in the synthesis, degradation, and interconversion of DNA, RNA, lipids, and carbohydrates. Pathogenic bacteria can use pyrimidine metabolism to potentially alter the metabolic activity of the hosts and create favourable conditions for themselves [73], and therefore affect the health of dental tissues.

Salivary epinephrine levels are correlated with the severity of periodontitis [74]. Moreover, enrichment analysis has demonstrated the involvement of increased epinephrine levels in the cAMP signalling pathway, which can regulate salivary amylase secretion [75, 76]. Amylase secretion contributes to reduced plaque acid produced by *Streptococcus mutans*, which dissolves the enamel and may be a biomarker for dental caries [77]. α-amylase is closely associated with dental caries; additionally, low α-amylase levels may promote the development of early childhood caries [78]. We observed upregulated levels of stachydrine, which has anti-inflammatory activity. In many Middle Eastern and African countries, Salvadora persica L. (toothbrush tree, Miswak) is used as a toothbrush, with its root being rich in stachydrine [79].

The remaining metabolic pathways such as glycerolipid metabolism and neuroactive ligand-receptor interaction are crucially involved in the pathogenesis of oral squamous carcinoma [80, 81]. Future studies should investigate their relationship with dental caries in children. We used ROC curves to assess the accuracy of salivary metabolites as biomarkers. In our study, we identified four metabolites that could be jointly used as biomarkers for SECC.

This study demonstrated that non-differential salivary microorganisms were related to caries severity and were mostly in the low-abundance species groups. This could be attributed to the following factors. First, saliva is not the site of caries occurrence; accordingly, caries occurrence is weakly correlated with salivary microorganisms. Second, our microbial sequencing depth may not have been sufficiently deep, and it would be better to draw conclusions at the species or strain level. Third, there is extensive heterogeneity in our ECC classification with respect to caries severity and intraoral distribution [82]. Further clarification of microbial roles should apply a combination of multi-omic approaches, including transcriptomics. The combined application of multi-omics may provide the most powerful diagnostic tool in studies on diseases [83].

Regarding metabolites, some differential metabolites were correlated with clinical data, which suggests the potential utility of salivary metabolites in dental caries research. Combined analysis of microorganisms and metabolites revealed significant correlations of most differential salivary microorganisms with metabolites. Specifically, *Veillonella* and *Staphylococcus* enriched in the SECC group as well as *Neisseria* and *Porphyromonas* enriched in the CF group were extensively correlated with metabolites. Most genera enriched in the SECC group were positively and negatively correlated with up-regulated and down-regulated metabolites, respectively, in the CF group. Opposite correlations were observed between genera enriched in the CF group and metabolites upregulated in the SECC group. Our findings confirm that host and oral microorganisms are closely connected and interact in the development of dental caries.

### Shortcomings and outlook

This study has several limitations. First, this study had a small sample size. Second, the depth of microbiome sequencing was not sufficiently deep; moreover, 16SrRNA technology could not sufficiently reveal the structure of flora composition under the species classification. Third, we did not conduct a longitudinal analysis. Future longitudinal studies combining host genomics, behavioural factors, and environmental factors, as well as screening of precise biomarkers, are warranted.

Using a multi-omics approach can help elucidate the composition and function of the salivary microbial community in the caries condition, as well as inform caries prevention and treatment.

## Supplementary Information

**Additional file 1**. 16S rRNA Gene Sequencing.

**Additional file 2: Fig. S1.** Taxon abundances at the phylum levels were compared between the SECC and CF groups using Metastats.

**Additional file 3: Fig. S2**. The random forest model was constructed for the genus taxonomic level (a). Comparison of model performance of random forests with different numbers of species, with the largest ROC values obtained for the 20 species selected (b). The AUC (Area Under Curve) is defined as the area under the ROC curve. Typically, it has a value between 1.0 and 0.5. For AUC > 0.5, the closer the AUC is to 1, the better the classification prediction is.

**Additional file 4. Fig. S3**. OPLS-DA 200 permutation testing. The R2Y(cum) and Q2(cum) results were (0.292, 0.131). The calculated R2X and R2Y(cum) estimates the goodness of fit of the model; Q2(cum) estimates the ability of prediction. For OPLS-DA, the permutation analysis between one predictive(p1) and three orthogonal (o1, o2, and o3) components produced the observed and cross-validated R2X, R2Y, and Q2 coefficients.

**Additional file 5. Fig. S4**. Correlations between microbiota (phylum level) and metabolites in saliva. Each row and column in the graph represents a metabolite and phylum, respectively, while each lattice represents a correlation coefficient between a component and a metabolite. Red and blue represent positive and negative correlations, respectively. * indicates a significant correlation between the phyla and metabolites (*p < 0.05, **p < 0.01).

## Data Availability

The datasets presented in this study can be found in SRA (Accession: PRJNA868496). https://www.ncbi.nlm.nih.gov/bioproject.
