# Supplementary material for: Salivary microbiome and metabolome analysis of severe early childhood caries
Source: BMC Oral Health. 2023 Jan 19;23:30. doi: 10.1186/s12903-023-02722-8 (PMC9850820; doi:10.1186/s12903-023-02722-8)
Supplement: Supplementary file 1 — Additional file 1. 16S rRNA Gene Sequencing. [file 12903_2023_2722_MOESM1_ESM.docx]

**Supplementary Fig. S1 Taxon abundances at the phylum levels were compared between the SECC and CF groups using Metastats.**

**Supplementary Fig. S2 The random forest model was constructed for the genus taxonomic level (a). Comparison of model performance of random forests with different numbers of species, with the largest ROC values obtained for the 20 species selected (b). The AUC (Area Under Curve) is defined as the area under the ROC curve. Typically, it has a value between 1.0 and 0.5. For AUC > 0.5, the closer the AUC is to 1, the better the classification prediction is.**

**Supplementary Fig. S3 OPLS-DA 200 permutation testing. The R2Y(cum) and Q2(cum) results were (0.292, 0.131). The calculated R2X and R2Y(cum) estimates the goodness of fit of the model; Q2(cum) estimates the ability of prediction. For OPLS-DA, the permutation analysis between one predictive(p1) and three orthogonal (o1, o2, and o3) components produced the observed and cross-validated R2X, R2Y, and Q2 coefficients.**

**Supplementary Fig. S4 Correlations between microbiota (phylum level) and metabolites in saliva. Each row and column in the graph represents a metabolite and phylum, respectively, while each lattice represents a correlation coefficient between a component and a metabolite. Red and blue represent positive and negative correlations, respectively. * indicates a significant correlation between the phyla and metabolites (**p* < 0.05, ***p* < 0.01).**

**16S rRNA Gene Sequencing**

1. Sequencing data processing

The reads of each sample were spliced using FLASH (V1.2.7, [http://ccb.jhu.edu/software/FLASH/)[1]](http://ccb.jhu.edu/software/FLASH/)%5b1%5d)after truncating the barcode and primer sequences, and the resulting spliced sequences were the original The spliced Raw Tags were then filtered to obtain high-quality Tags (Clean Tags)[2]. Referring to the Tags quality control process in Qiime (V1.9.1, http://qiime.org/scripts/split_libraries_fastq.html)[3], the following operations were performed: a) Tags interception: Raw Tags were intercepted from consecutive low-quality values (default quality threshold <=19) to a set number of bases (default length value is 3); b) Tags length filtering: the Tags data set obtained after interception is further filtered to remove Tags with consecutive high-quality bases less than 75% of the Tags length. The Tags obtained after the above process need to be processed to remove the chimeric sequences, and the Tags sequences are compared with the species annotation database by (https://github.com/torognes/vsearch/)[4] to detect the chimeric sequences and finally remove the chimeric sequences from them to obtain the final effective data ( Effective Tags).

2. OTU clustering and species annotation

All Effective Tags from all samples were clustered using the Uparse algorithm (Uparse v7.0.1001, http://www.drive5.com/uparse/)[5]. Sequences with ≥97% similarity were assigned to the same operational taxonomic units (OTUs). The representative sequences of OTUs are selected based on the principles of the algorithm, and the sequences with the highest frequency of occurrence in OTUs are chosen as representative sequences of OTUs. Species annotation of OTUs sequences was analyzed using the Mothur method with the SSUrRNA database [6] from SILVA138 (http://www.arb-silva.de/) [7] for species annotation (setting a threshold of 0.8 to 1) to obtain taxonomic information and separate statistics on the community composition of each sample at each taxonomic level: kingdom, phylum, class, order, family, genus, species. phylum, class, order, family, genus, species, and the community composition of each sample. A multiple sequence alignment was performed using MUSCLE [8] (Version 3.8.31, http://www.drive5.com/muscle/) software to obtain phylogenetic relationships for all OTUs representative of the sequences. Finally, The abundance information of the OTUs was normalized using a standard of sequence number corresponding to the sample with the least number of sequences. The subsequent computation of alpha and beta diversities was performed using QIIME (Version 1.9.1).

References

1. Magoč, Tanja, and Steven L. Salzberg. FLASH: fast length adjustment of short reads to improve genome assemblies. Bioinformatics 27.21 (2011): 2957-2963.
2. Bokulich, Nicholas A., et al. Quality-filtering vastly improves diversity estimates from Illumina amplicon sequencing. Nature methods 10.1 (2013): 57-59.
3. Caporaso, J. Gregory, et al. QIIME allows analysis of high-throughput community sequencing data. Nature methods 7.5 (2010): 335-336.
4. Rognes T, Flouri T, Nichols B, Quince C, Mahé F. (2016) VSEARCH: a versatile open source tool for metagenomics. PeerJ 4:e2584 <https://doi.org/10.7717/peerj.2584.>
5. Haas, Brian J., et al. Chimeric 16S rRNA sequence formation and detection in Sanger and 454-pyrosequenced PCR amplicons. Genome research 21.3 (2011): 494-504.
6. Wang, Qiong, et al. Naive Bayesian classifier for rapid assignment of rRNA sequences into the new bacterial taxonomy. Applied and environmental microbiology 73.16 (2007): 5261-5267.
7. Edgar, Robert C. UPARSE: highly accurate OTU sequences from microbial amplicon reads. Nature methods 10.10 (2013): 996-998.
8. Quast C, Pruesse E, et al.The SILVA ribosomal RNA gene database project: improved data processing and web-based tools. Nucl. Acids Res. (2013) : D590-D596.
