# Supplementary figures and images for: Salivary microbiome and metabolome analysis of severe early childhood caries
Source: BMC Oral Health. 2023 Jan 19;23:30. doi: 10.1186/s12903-023-02722-8 (PMC9850820; doi:10.1186/s12903-023-02722-8)

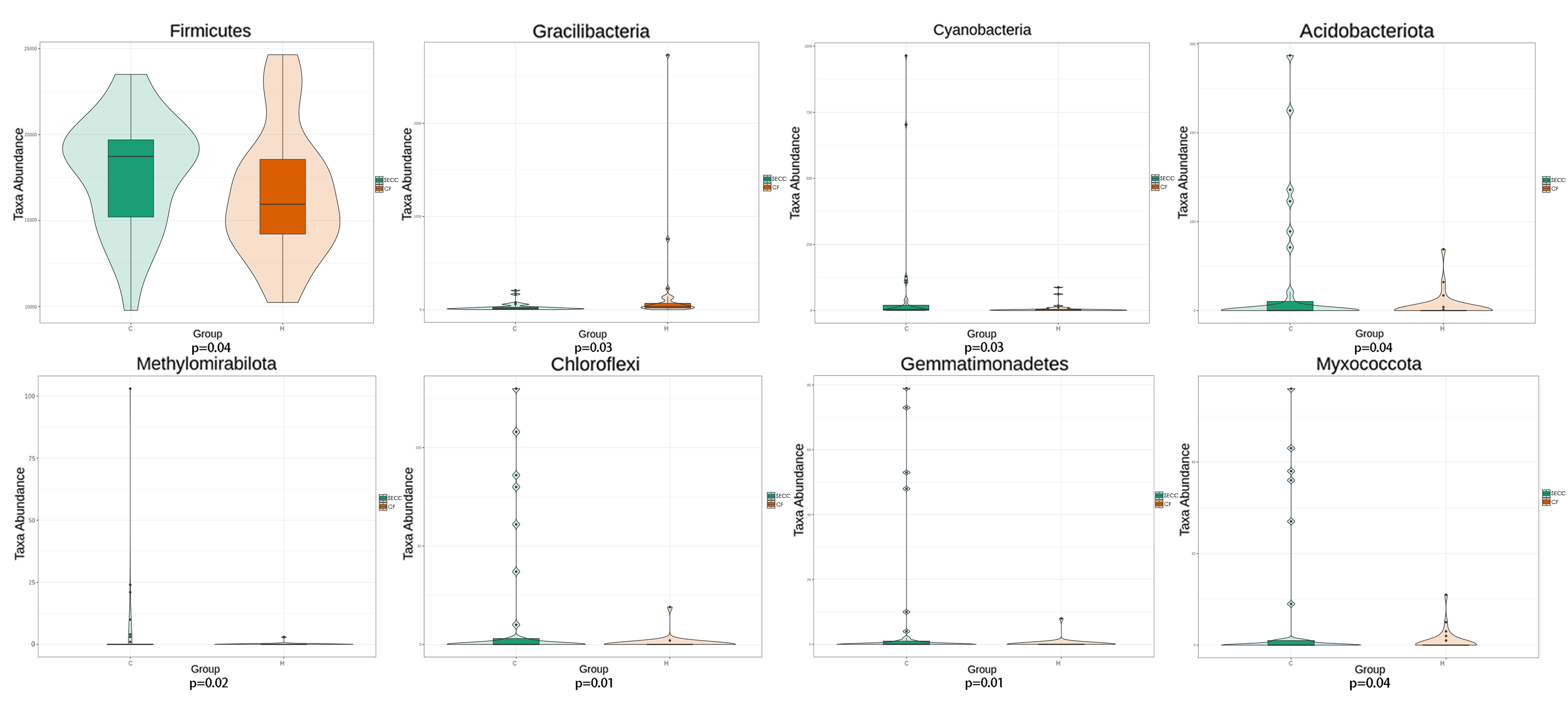

Supplement: Supplementary file 2 — Additional file 2: Fig. S1. Taxon abundances at the phylum levels were compared between the SECC and CF groups using Metastats. [file 12903_2023_2722_MOESM2_ESM.tif]

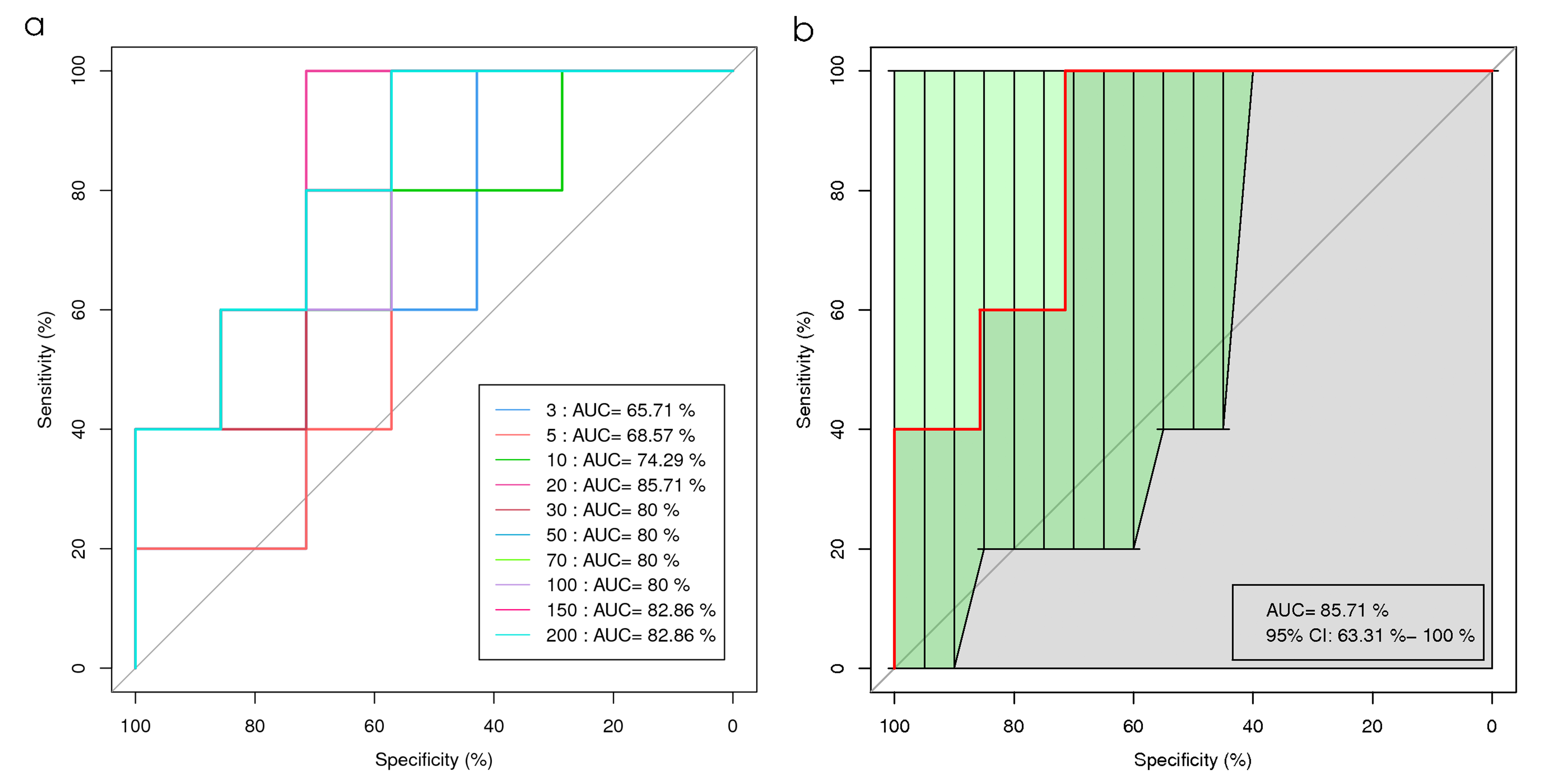

Supplement: Supplementary file 3 — Additional file 3: Fig. S2. The random forest model was constructed for the genus taxonomic level (a). Comparison of model performance of random forests with different numbers of species, with the largest ROC values obtained for the 20 species selected (b). The AUC (Area Under Curve) is defined as the area under the ROC curve. Typically, it has a value between 1.0 and 0.5. For AUC > 0.5, the closer the AUC is to 1, the better the classification prediction is. [file 12903_2023_2722_MOESM3_ESM.tif]

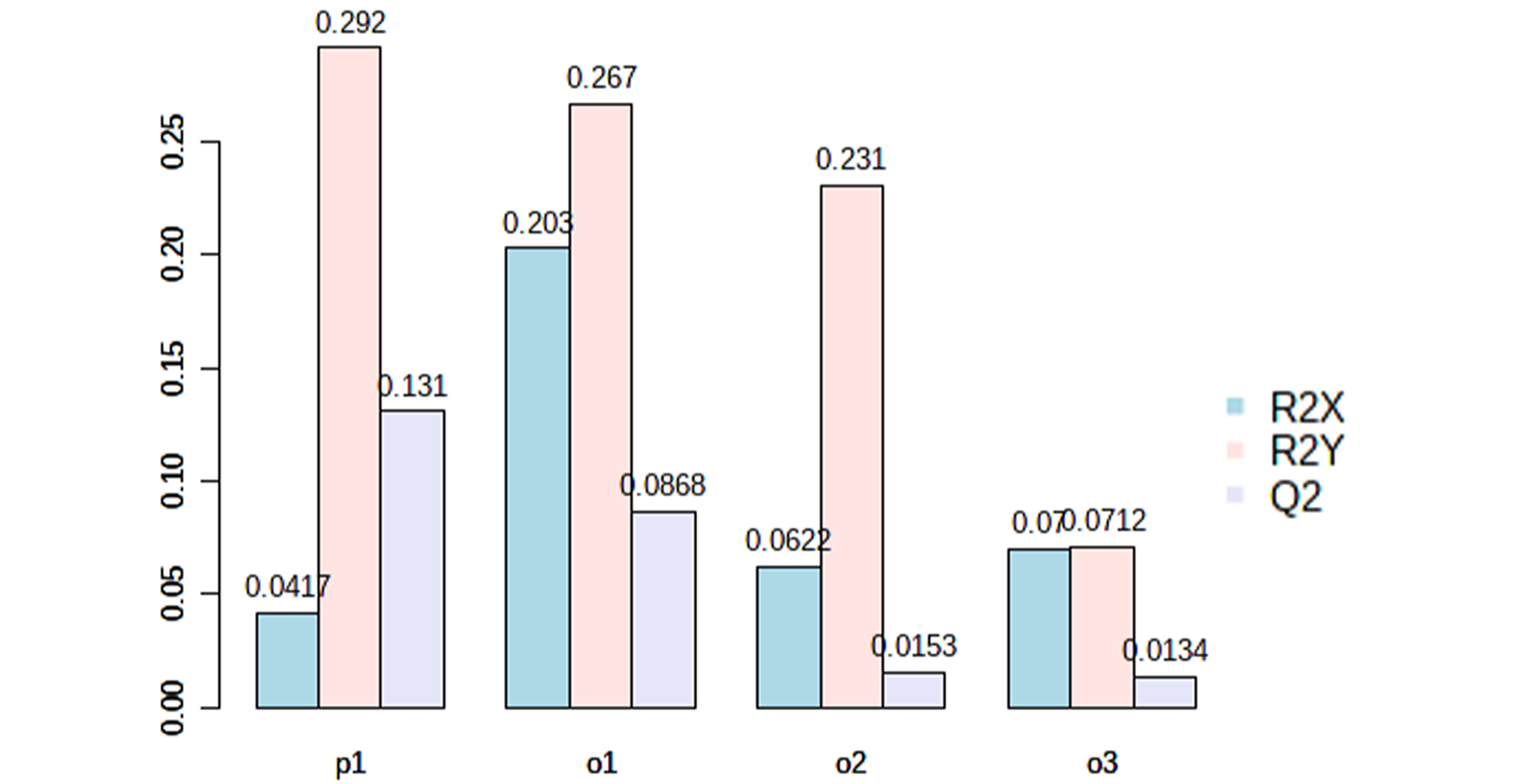

Supplement: Supplementary file 4 — Additional file 4. Fig. S3. OPLS-DA 200 permutation testing. The R2Y(cum) and Q2(cum) results were (0.292, 0.131). The calculated R2X and R2Y(cum) estimates the goodness of fit of the model; Q2(cum) estimates the ability of prediction. For OPLS-DA, the permutation analysis between one predictive(p1) and three orthogonal (o1, o2, and o3) components produced the observed and cross-validated R2X, R2Y, and Q2 coefficients. [file 12903_2023_2722_MOESM4_ESM.tif]

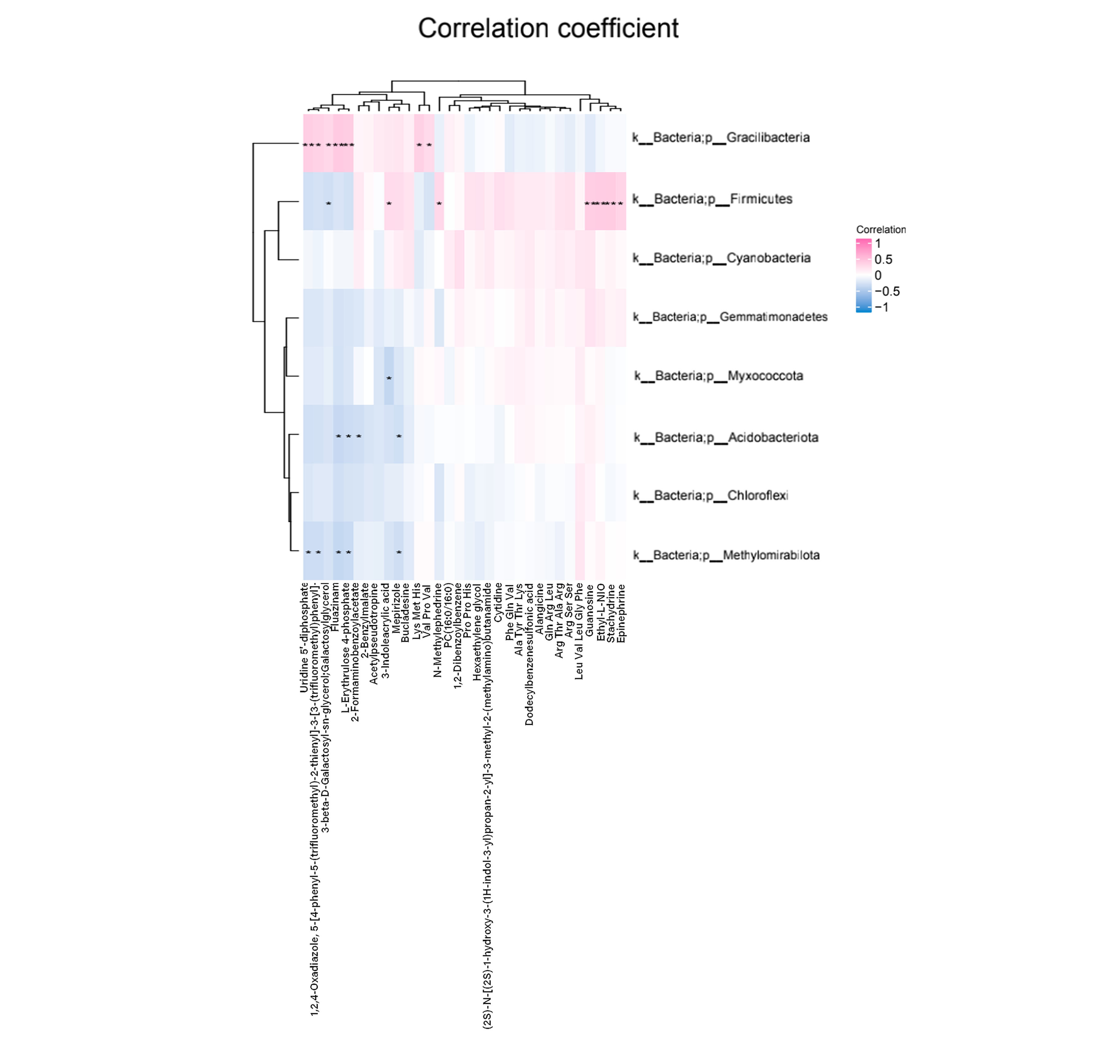

Supplement: Supplementary file 5 — Additional file 5. Fig. S4. Correlations between microbiota (phylum level) and metabolites in saliva. Each row and column in the graph represents a metabolite and phylum, respectively, while each lattice represents a correlation coefficient between a component and a metabolite. Red and blue represent positive and negative correlations, respectively. * indicates a significant correlation between the phyla and metabolites (*p < 0.05, **p < 0.01). [file 12903_2023_2722_MOESM5_ESM.tif]
